# Were surgical outcomes for acute appendicitis impacted by the COVID-19 pandemic?

**DOI:** 10.1186/s12893-023-01930-x

**Published:** 2023-02-23

**Authors:** Rachel Waldman, Harrison Kaplan, I. Michael Leitman

**Affiliations:** Department of Surgery, Icahn School of Medicine at Mount Sinai, One Gustave L. Levy Place, Box 1076, New York, NY 10029 USA

**Keywords:** Appendicitis, COVID-19, Outcomes, Pandemic, Resilience

## Abstract

**Background:**

The COVID-19 pandemic disrupted healthcare systems throughout the world. We examine whether appendectomy outcomes in 2020 and 2021 were affected by the pandemic.

**Methods:**

We conducted a retrospective cohort study of 30-day appendectomy outcomes using the ACS-NSQIP database from 2019 through 2021. Logistic regression and linear regression analyses were performed to create models of post-operative outcomes.

**Results:**

There were no associations between the time period of surgery and death, readmission, reoperation, deep incisional SSI, organ space SSI, sepsis, septic shock, rate of complicated appendicitis, failure to wean from the ventilator, or days from admission to operation. During the first 21 months of the pandemic (April 2020 through December 2021), there was a decreased length of hospital stay (p = 0.016), increased operative time (p < 0.001), and increased likelihood of laparoscopic versus open surgery (p < 0.001) in compared to 2019.

**Conclusions:**

There were minimal differences in emergent appendectomy outcomes during the first 21 months of the pandemic when compared to 2019. Surgical systems in the US successfully adapted to the challenges presented by the COVID-19 pandemic.

## Background

On March 11th, 2020, the World Health Organization officially declared the coronavirus disease 2019 (COVID-19) a pandemic, marking the start of an unprecedented period of worldwide social, economic, and healthcare disruptions [1]. The landscape of patient care abruptly changed as hospitals restructured to accommodate the flood of COVID-19 patients and to minimize the risk of exposure to healthcare providers and other patients [2]. The American College of Surgeons (ACS) recommended the rescheduling of elective procedures [3, 4], and surgical services contended with concerns regarding the availability of personal protective equipment (PPE), ICU beds, personnel, and other hospital resources, as well as potential COVID-19 exposure through electrocautery smoke and laparoscopic techniques [2, 5]. Surgical services had to adopt evolving COVID-19 pre-procedure testing protocols, and at times, urgent operations were delayed as it became clear that procedures performed on patients with active COVID-19 infection had a higher morbidity and mortality [6]. Surgeons and hospitals were forced to quickly adapt to new protocols to make the safest and best decisions for patients in the rapidly evolving pandemic landscape, and many of these new protocols have continued to evolve over since the start of the pandemic, with many COVID-19 changes now permanently part of hospital practice.

Emergent surgeries continued throughout the pandemic, including appendectomies to treat acute appendicitis. Acute appendicitis is one of the most common indications for emergency general surgery throughout the world [7, 8]. The standard of care for acute appendicitis is generally considered to be laparoscopic appendectomy, although with some consideration of antibiotic therapy as a potential treatment for certain indications [9–12]. There has been significant interest surrounding the effects of the COVID-19 pandemic on volume, severity, and treatment of acute appendicitis, with evidence supporting an increase in the rate of antibiotic treatment for acute appendicitis during the pandemic as well as higher rates of complicated and perforated appendicitis observed in adults [10, 13–18]. Additionally, as society continues to adapt to live with COVID as a potentially permanent part of the healthcare landscape, there has been little research into how the continued adaptions around COVID-19 affect how surgical systems function today.

The current article evaluates whether emergent laparoscopic and open appendectomy outcomes in April-June 2020 were affected by the rapid changes and stressors posed by the beginning of the COVID-19 pandemic, and whether emergent laparoscopic and open appendectomy outcomes since the onset of pandemic in March 2020 through 2021 differ from outcomes pre-pandemic using the American College of Surgeons—National Surgical Quality Improvement Database (ACS-NSQIP). Understanding emergency surgical outcomes in the United States during both the acute phase of the COVID-19 pandemic and continued through 2021 might assess how emergency surgical services would react and adapt to potential future system-wide stressors of the healthcare system as well as how lasting changes from the COVID-19 pandemic continue to affect surgical systems in the United States today.

## Methods

This was a retrospective cohort study using the ACS-NSQIP database from the years 2019, 2020 and 2021. The ACS-NSQIP database consists of de-identified anonymized datasets of 30-day surgical outcomes for about 700 participating hospitals; data is collected at each hospital by a surgical clinical reviewer who accesses a random sample of patients [19]. The database was obtained from ACS-NSQIP participant user files (PUF) available to all member hospitals by submitting a request form to the following link (https://www.facs.org/quality-programs/data-and-registries/acs-nsqip/participant-use-data-file/participant-use-request-form/). We selected patients who underwent an emergent laparoscopic or open appendectomy by identifying patients having undergone primary operation with a current procedural terminology (CPT) code of 44,950, 44,960, or 44,970. Demographics, comorbidities, clinical data, and complications were collected. Patients with incomplete data for baseline characteristics and outcomes including age, American Society of Anesthesiology (ASA) score, length of stay, days to operation, preoperative white blood cell count, and total operative time, were excluded.

This study was reviewed by the Institutional Review Board of the Icahn School of Medicine at Mount Sinai in New York, NY including a waiver of patient consent. The American College of Surgeons National Surgical Quality Improvement Program and the hospitals participating in the ACS NSQIP are the source of the data used herein; they have not verified and are not responsible for the statistical validity of the data analysis or the conclusions derived by the authors.

The data were separated into a pre-COVID interval (January 1st 2019 through December 31st 2019) and an interval over 21 months of the pandemic (April 1st 2020 through December 31st 2021). The NSQIP dataset only makes available admission year and quarter, and as COVID-19 was officially declared a pandemic by the World Health Organization in March 2020, the first quarter of 2020 (January 1st through March 31st 2020) includes both pre- and post-pandemic data that could not be separated and was excluded from our study. The dataset was also separated into an initial pandemic period, defined as the second quarter of 2020 (January 1st 2020 through June 31st 2020) and a comparison pre-COVID timeframe, the second quarter of 2019 (April 1st 2019 through June 31st 2019), to assess the more immediate impact of COVID-19 and to account for any potential seasonal variation. Baseline characteristics for each of these time periods were analyzed. We then used chi-squared tests to determine variables that were significantly associated with complications and other characteristics available in the NSQIP database including laparoscopic versus open surgery, complicated appendicitis, death, readmission, sepsis, septic shock, failure to wean from ventilator within 48 h, transfusions, Deep Incisional Surgical Site Infections (SSI), Organ Space SSI, and reoperation. Complicated appendicitis was defined as having one of the following primary International Classification of Diseases (ICD) 10th revision codes: K35.20, K35.21, K35.31, K35.32, K35.33, or K35.891. For each timeframe comparison, logistic regression was then performed to create a model for each of the above binary complications, using the demographic and comorbidity variables that had significant associations on univariate analysis as well as the time period. Similarly, linear regression was used to create models for continuous outcomes including the total length of stay, days from admission to operation, preoperative white blood cell count, and total operative time. The variables used in the final models were time period, gender, age of 65 or greater, current smoker, dyspnea, ascites, congestive heart failure, hypertension, renal failure, dialysis, disseminated cancer, steroid/immunosuppressant therapy, malnourishment, bleeding disorder, body mass index (BMI) greater than 30, and ASA score of 3 or greater.

The c-statistic and normalized root mean squared error (RMSE) were used to assess the fit of the logistic and linear regression models, respectively. Normalized RMSE was calculated by dividing the RMSE by the range of observed values of the outcome being predicted.

## Results

There were 27,889 patients with complete records in the database over the course of 2019, and 42,811 patients with complete records in the pandemic timeframe (April 1st 2020 through December 31st 2021). Of these 70,700 total patients, 68,011 patients underwent a laparoscopic appendectomy, while the remaining 2689 underwent an open appendectomy. Patient demographics were largely consistent across timeframes studied, as seen in Tables 1 and 2. There were no associations between the time period of surgery and death, readmission, reoperation, deep incisional SSI, organ/space SSI, sepsis, septic shock, rate of complicated appendicitis, failure to wean from the ventilator, or days from admission to operation. Receiving an appendectomy during the pandemic (April 2020 through December 2021) was a predictor of decreased length of hospital stay (average of 1.77 days pre-pandemic versus 1.74 days during the pandemic, p = 0.016), increased operative time (average of 49.76 min pre-pandemic versus 50.75 min during the pandemic, p < 0.001), and likelihood of laparoscopic rather than open surgery (4.36% pre-pandemic versus 3.44% during the pandemic, p < 0.001). Operation during the early pandemic, between April 1st 2020 and June 31st 2020, when compared to the same time period in 2019, was associated with an increased preoperative white blood cell count (13.12 in quarter 2 of 2019 versus 13.26 in quarter 2 of 2020, p = 0.033), longer operative time (49.12 min in quarter 2 of 2019 versus 50.33 in quarter 2 of 2020, p = 0.032), and increased likelihood of laparoscopic rather than open surgery (4.14% in quarter 2 of 2019 versus 4.07% in quarter 2 of 2020, p < 0.001). The c-statistic of the logistic regression models ranged from 0.590 to 0.945 as shown in Table 3***.*** The normalized RMSE of our linear regression models ranged from 0.013 to 0.090.

**Table 1:** Comparison of Baseline Characteristics between Quarter 2 of 2019 and Quarter 2 of 2020

	2019 Quarter 2 (n = 6,795)	2020 Quarter 2 (n = 7,264)	*p*-value
	Mean	Mean	
Age	40.07	40.15	0.192
BMI	25.22	24.31	< 0.001
ASA	1.88	1.99	0.044

	2019 Quarter 2 (n = 6,795)	2020 Quarter 2 (n = 7,264)	*p*-value
	n (%)	n (%)	
Female	3,337 (49.11)	3,604 (49.61)	0.550
White	4,053 (59.65)	3,845 (52.93)	< 0.001
Black	403 (5.93)	385 (5.30)	0.104
Other Race	460 (6.77)	449 (6.18)	0.156
Hispanic	1,040 (15.31)	1,032 (14.21)	0.066
Diabetic	121 (1.78)	149 (2.05)	0.243
Cigarette Use	1,005 (14.79)	1,056 (14.54)	0.672
Dyspnea	91 (1.34)	83 (1.14)	0.292
Ventilator Dependent	6 (0.09)	1 (0.01)	0.048
Independent	6,726 (98.98)	7,196 (99.06)	0.632
Partially Dependent	19 (0.28)	24 (0.33)	0.586
COPD	66 (0.97)	66 (0.91)	0.700
CHF	12 (0.18)	18 (0.25)	0.361
Hypertension	1,051 (15.47)	1,058 (14.56)	0.134
Renal Failure	10 (0.15)	9 (0.12)	0.707
Dialysis	15 (0.22)	12 (0.17)	0.452
Disseminated Cancer	12 (0.18)	22 (0.30)	0.128
Open Wound	8 (0.12)	4 (0.06)	0.204
Steroid Use	93 (1.37)	92 (1.27)	0.595
Weight Loss	12 (0.19)	9 (0.12)	0.312
Bleeding Disorder	88 (1.30)	118 (1.62)	0.104

*BMI* Body Mass Index, *ASA* American Society of Anesthesiology Score, *COPD* Chronic Obstructive Pulmonary Disease, *CHF* Congestive Heart Failure

**Table 2:** Comparison of baseline characteristics between 2019 and April, 1 2020 through December 31, 2021

	2019 (n = 27,889)	April 2020–December 2021 (n = 19,269)	p-value
	Mean	Mean	
AGE	39.91	40.15	0.184
BMI	25.08	24.70	0.002
ASA	1.91	1.96	0.040

	2019 (n = 27,889)	April 2020–December 2021 (n = 19,269)	p-value
	n (%)	n (%)	
Female	13,749 (49.30)	9,535 (49.48)	0.693
White	16,222 (58.17)	10,435 (54.15)	< 0.001
Black	1,647 (5.91)	1,155 (5.99)	0.690
Other Race	1,867 (6.69)	1,321 (6.86)	0.493
Hispanic	4,227 (15.16)	2,758 (14.31)	0.011
Diabetic	568 (2.04)	408 (2.12)	0.545
Cigarette Use	4,216 (15.12)	2,767 (14.36)	0.023
Dyspnea	354 (1.27)	243 (1.26)	0.937
Ventilator Dependent	12 (0.04)	7 (0.04)	0.722
Independent	27,554 (98.80)	19,105 (99.15)	< 0.001
Partially Dependent	97 (0.35)	59 (0.31)	0.439
COPD	270 (0.97)	174 (0.90)	0.472
CHF	51 (0.18)	39 (0.20)	0.633
Hypertension	4,210 (15.10)	2,807 (14.57)	0.113
Renal Failure	39 (0.14)	32 (0.17)	0.470
Dialysis	62 (0.22)	38 (0.20)	0.560
Disseminated Cancer	47 (0.17)	50 (0.26)	0.032
Open Wound	33 (0.12)	14 (0.07)	0.122
Steroid Use	363 (1.30)	249 (1.29)	0.930
Weight Loss	39 (0.14)	31 (0.16)	0.560
Bleeding Disorder	388 (1.39)	294 (1.53)	0.229

*BMI* Body Mass Index, *ASA* American Society of Anesthesiology Score, *COPD* Chronic Obstructive Pulmonary Disease, *CHF* Congestive Heart Failure

**Table 3:** Associations between outcomes following appendectomy

	Quarter 2				2019 vs. April 2020 – December 2021			
		2019 (n = 6,795)	2020 (n = 7,264)			Pre (n = 27,889)	Post (n = 42,811)	
	p-value	n (%)	n (%)	C Statistic	p-value	n (%)	n (%)	C Statistic
Open	< 0.001	281 (4.14)	296 (4.07)	0.622	< 0.001	1,217 (4.36)	1,472 (3.44)	0.647
Complicated	0.599	901 (13.26)	940 (12.94)	0.602	0.090	3,563 (12.78)	5,944 (13.88)	0.605
Deaths	0.525	6 (0.09)	5 (0.07)	0.945	0.542	25 (0.09)	40 (0.09)	0.935
Readmissions	0.291	259 (3.81)	310 (4.27)	0.599	0.529	1,066 (3.82)	1,677 ( 3.92)	0.590
Reoperations	0.792	65 (0.96)	73 (1.00)	0.601	0.878	277 (0.99)	393 (0.92)	0.618
Wound Infections	0.418	9 (0.13)	6 (0.08)	0.937	0.695	50 (0.18)	63 (0.15)	0.669
Organ Space SSI	0.102	244 (3.59)	225 (3.10)	0.599	0.535	930 (3.33)	1435 (3.35)	0.599
Sepsis	0.790	311 (4.58)	338 (4.65)	0.615	0.075	1,156 (4.15)	2,017 (4.71)	0.623
Septic Shock	0.294	16 (0.24)	25 (0.34)	0.860	0.588	95 (0.34)	154 (0.36)	0.880
Failure to wean	0.412	9 (0.13)	6 (0.08)	0.937	0.357	46 (0.16)	60 (0.14)	0.903
Transfusions	0.142	18 (0.26)	31 (0.43)	0.737	0.312	76 (0.27)	121 (0.28)	0.736

	Quarter 2				2019 vs. April 2020 – December 2021			
		2019 (n = 6,795)	2020 (n = 7,264)			Pre (n = 27,889)	Post (n = 42,811)	
		Mean		Norm. RMSE		Mean		Norm. RMSE
Days to Operation	0.126	0.30	0.34	0.014	0.054	0.31	0.34	0.013
Length of Stay	0.186	1.76	1.72	0.027	0.016	1.77	1.74	0.025
Operative Time	0.032	49.12	50.33	0.036	< 0.001	49.76	50.75	0.027
White Blood Cells	0.033	13.12	13.26	0.090	0.076	13.04	13.09	0.090

## Discussion

There were very few differences in outcomes following emergent laparoscopic and open appendectomy during the COVID-19 pandemic compared to the year prior to the pandemic among hospitals participating in the ACS-NSQIP. The NSQIP dataset, a prospective, validated dataset using 30-day outcome data from about 700 hospitals, provides a large dataset with high internal validity, allowing for an analysis of how 30-day appendectomy outcomes vary by timeframe throughout the United States with greater statistical power than previous studies on the topic. Previous studies using the ACS-NSQIP database have shown seasonal variations in outcomes after surgery [20], and this is compounded by the varying waves of the COVID-19 pandemic throughout 2020. By comparing quarter 2 of 2020 with the same quarter in 2019, and by comparing combined data from all of 2019 with outcomes from April 2020 through December 2021, we attempted to minimize the effects of these variations. We compared a variety of 30-day post-surgical outcomes including death, readmission, deep incisional SSI, organ space SSI﻿, prolonged ventilator requirement, sepsis, shock, and reoperations during the acute beginning of the COVID-19 pandemic (April 1st 2020 through June 31st 2021) to the corresponding time frame of 2019 in order to understand the effects of the early pandemic on surgical outcomes, and we additionally compared these same variables between the entirety of 2019 and an extended pandemic timeframe of April 1st 2020 through December 31st 2021, capturing 21 months of systematic adaptation to COVID-19. There were no significant differences in surgical outcomes during the first 21 months of the pandemic when compared to 2020, and similarly there were no differences in outcomes in the second quarter of 2020, during the early phases of the COVID-19 pandemic, when compared to the corresponding quarter of 2019. While there were minimal differences in long-term outcomes, there were significant differences noted in variables related to the immediate perioperative period including in method of surgery (open vs laparoscopic). Overall, this study demonstrates the resiliency of surgical systems throughout the United States. Amid disruptions in workflow, limited PPE and available hospital beds, and the threat of contracting and spreading COVID-19, during April through July of 2020, surgeons and the systems they function in were able to maintain surgical outcomes consistent with those observed pre-pandemic. As our surgical systems continue to adapt to a world where COVID-19 seems to be here to stay, appendectomy outcomes during the first 21 months of the continued pandemic were consistent with pre-pandemic outcomes indicating a sustainable adaptation of the surgical system to new practices and norms that may be slowly becoming permanent aspects of our healthcare landscape.

In addition to binary complications and outcomes, we compared other data points and metrics including open vs laparoscopic approach, length of stay, days from admission to operation, preoperative white blood cell count, and total operative time. During the early initial stages of the pandemic, there was brief concern that COVID-19 could be spread though aerosolization of the virus during laparoscopic surgery. The ACIE study, which surveyed 709 surgeons from 66 countries in early April 2020, found that, in the absence of clear evidence or guidance, one-third of surgeons surveyed reported changing from a laparoscopic to open surgical approach [16]. While our study cannot comment specifically on March or April 2020, a comparison of April 1st through June 31st 2020 to the same timeframe in 2019 demonstrates a higher percentage of laparoscopic surgeries than open surgeries. While this very small decrease in open appendectomies from 4.14 to 4.07% of cases is likely a more statistically significant than clinically significant decrease, this indicates that perhaps concern for viral aerosolization of COVID-19 did not in fact lead to significant or sustained change in surgical approach in the United States even during the early phases of the pandemic. Furthermore, a laparoscopic approach was found to be more frequent during the first 21 months of the pandemic when compared to 2019, a finding consistent with the continued trend towards laparoscopic appendectomy rather than open appendectomy as the standard of care [21, 22].

Operative time was found to be significantly increased in the second quarter of 2020 when compared to the same time period of 2019 as well as during the first 21 months of the pandemic when compared to the entirety of 2019. However, in both comparisons the difference in mean operative time was under 1.5 min, and thus this finding may not in fact be clinically significant, particularly considering the absence of an associated difference in outcomes. Length of stay was also found to be slightly shorter during the first 21 month of the pandemic when compared to 2019, with the average length of stay decreasing from 1.77 days to 1.74 days. While this finding was statically significant, this decrease is so minimal that it likely is not clinically meaningful. It is perhaps worth noting though that laparoscopic appendectomy has been associated with a shortened length of stay in previous studies, and thus this decrease in length of stay case may be related to increased use of a laparoscopic rather than open approach [22, 23].

During the early stages of the pandemic, there were concerns about potential adverse outcomes due to patient’s delaying arrival to the hospital due to fear of contracting COVID-19 in a healthcare setting as well as the possibility of in-hospital delays due to waiting for PCR testing results to return, strained hospital resources, additional precautions by staff to avoid exposure, and continually evolving infection prevention protocols. We found that there was no significant difference in time from admission to operation in either the initial COVID period (April 2020–June 2020) or the first 21 months of the pandemic when compared to 2019. 

Previous research has suggested an increase in cases of complicated appendicitis during the early stages of the COVID-19 pandemic, perhaps a consequence of delayed presentation due to fear of contracting COVID-19 in hospitals [10, 13, 14, 24]. An international meta-analysis found 21 studies evaluating the rate of complicated appendicitis (involving abscess, perforation, or peritonitis). 7474 patients during the pandemic were compared to 18,107 cases before the pandemic, with 26.6% of patients having complicated appendicitis during the pandemic compared to just 22.2% before the pandemic [10]. In contrast, in the present study, there was no change in the frequency of complicated appendicitis in either time frame studied when compared to 2019, suggesting that increased complicated appendicitis cases in the setting of delays in care early in the pandemic may have been less prevalent than previously assumed. When examining this data, it is interesting to consider that non-operative, antibiotic-only management of uncomplicated appendicitis increased over the course of the pandemic [4, 10, 16, 18, 25]. An increase in non-operative, antibiotic-only management of appendicitis would result in these, often uncomplicated, cases of appendicitis being no longer included in this appendectomy outcome data, with potentially later surgical intervention in setting of some patients failing conservative treatment resulting in progression of appendicitis. However, there were no significant differences in surgical outcomes, no statistically significant difference in days from admission to operation, and no statistically significant increase in cases of complicated appendicitis during April 1st 2020 through December 31st 2021 when compared to 2019. While there are many possible explanations for these outcomes, it does not appear to have resulted in patients’ progression to complicated appendicitis or complex operative procedures. While our dataset only allows for analysis of operative cases of appendicitis, there may be opportunities for additional studies to determine the effects of increased antibiotic-only treatment of appendicitis and whether this trend continues in 2022 and onwards. Overall, the minimal difference in outcomes during the COVID-19 pandemic indicates that surgeons were able to maintain consistent outcomes regardless of any protocol changes, demonstrating the ability of surgical systems to adapt effectively despite increased systemic stressors.

This study has several important limitations that may affect the interpretation and generalizability of these findings. First, there are inherent limits to the ACS-NSQIP database—only a subset of hospitals participate in this database, and thus demographic differences between NSQIP-participating hospitals and non-participating hospitals may affect the generalizability of these findings. Several variables, particularly deaths and greater than 48 h on a ventilator post-op, were very rare outcomes, and due to these small effective sample sizes, there is not significant statistical power to conclude for certain that year of surgery did not impact these outcomes. While the NSQIP dataset has the advantage of its large scale, the de-identified nature of the data and absence of many clinical data points makes it more challenging to observe local trends and further analyze different sub-types of appendicitis. The COVID-19 pandemic evolved rapidly over the course of 2020, and affected different geographic regions at different times, thus both the local effects of COVID-19 and short-term effects (particularly events over a few week) may not be realized in this analysis of national data broken down quarterly. An additional consequence of this quarterly breakdown is that the first quarter of 2020 (January through March), has been excluded from the analysis as it includes both pre- and post-pandemic outcomes. Additionally, it’s important to recognize the uniqueness of the 2020 COVID-19 outbreak. One may not assume that the outcomes from this initial phase of the pandemic will be applicable to future COVID-19 waves or other potential disruptions to the surgical system.

## Conclusion

While elective surgeries were paused and urgent operations were delayed at many points during the COVID-19 pandemic, emergency surgeries, including appendectomies, continued, and as COVID-19 has become part of the continued healthcare landscape over the past two-and-a-half-years, surgical systems have continued to adopt new practices and protocols. In the present study using the ASC-NSQIP database, we found that there were minimal differences in the outcomes from emergent laparoscopic and open appendectomy during the early COVID-19 pandemic (April through June 2020), when compared to the prior year, and similarly minimal differences in outcomes and other metrics over the first 18 months of the COVID-19 pandemic (April 2020 through December 2021) when compared to 2019. While these findings might be limited by the analysis of only a single procedure type, they suggest that, overall, surgical systems in the US were able to successfully adapt to the variety of challenges presented by the ongoing evolution of the pandemic.

## Data Availability

The datasets used and analyzed during the current study are available the American College of Surgeons as part of the National Surgical Quality Improvement Database, but restrictions apply to the availability of these data, which were used under license for the current study, and so are not publicly available. The datasets generated and analyzed for the current study are available from the corresponding author on reasonable request.

## Abbreviations

ACS: American College of Surgeons
ASA Score: American Society of Anesthesiology Score
BMI: Body Mass Index
CPT Code: Current Procedural Terminology Code
COVID-19: Coronavirus Disease 2019
ICD Code: International Classification of Diseases Code
ICU: Intensive Care Unit
NSQIP: National Surgical Quality Improvement Database
PCR Test: Polymerase Chain Reaction Test
PPE: Personal Protective Equipment
RMSE: Root Mean Squared Error
SSI: Surgical Site Infection
WBC Count: White Blood Cell Count
